# Submental Flap Reconstruction for Mandibular Osteomyelitis in an Adolescent Patient With Osteopetrosis: A Case Report

**DOI:** 10.7759/cureus.116923

**Published:** 2026-09-25

**Authors:** Jeremi Matysek, Dominika Lech, Robert Maksymowicz, Cyprian Straczek, Krzysztof Dowgierd

**Affiliations:** 1 Department of Clinical Pediatrics, Head and Neck Surgery Clinic for Children and Young Adults, University of Warmia and Mazury in Olsztyn, Olsztyn, POL; 2 Department of Maxillofacial, Reconstructive and Plastic Surgery, Head and Neck Surgery Clinic for Children and Young Adults, Regional Specialized Children’s Hospital in Olsztyn, Olsztyn, POL

**Keywords:** clcn7 mutation, craniofacial reconstruction, jaw complications, maxillofacial surgery, osteopetrosis, pediatric patient, soft-tissue reconstruction, submental flap

## Abstract

Osteopetrosis is a rare group of skeletal disorders characterized by impaired osteoclastic bone resorption and abnormal bone remodeling. Severe forms may result in bone marrow failure, extramedullary hematopoiesis, cranial nerve dysfunction, and skeletal complications. Mandibular osteomyelitis is a particularly challenging complication because impaired bone remodeling and compromised vascularity may contribute to persistent infection, osteonecrosis, and poor wound healing. We report the case of a 14-year-old girl with severe osteopetrosis, reported in her previous medical records as CLCN7-related, complicated by severe hematological abnormalities, hepatosplenomegaly, neurological complications, and chronic mandibular osteomyelitis with osteonecrosis. The patient underwent surgical removal of necrotic mandibular bone followed by reconstruction of the anterior floor-of-mouth soft-tissue defect using a submental flap. Preoperative computed tomography angiography confirmed patency of the relevant facial and submental vessels and supported the planned reconstruction. Given the patient's anemia, thrombocytopenia, malnutrition, and limited physiological reserve, a limited surgical approach was selected to avoid more extensive mandibular resection. Following surgery, significant postoperative airway edema required continued mechanical ventilation and pediatric intensive care unit management. After multidisciplinary postoperative care, successful extubation was achieved on the third postoperative day, followed by transfer to the maxillofacial surgical ward in a hemodynamically and respiratory stable condition with satisfactory early postoperative healing. This case highlights the challenges of managing mandibular osteomyelitis in patients with severe osteopetrosis and significant systemic disease. Individualized surgical planning and multidisciplinary perioperative management are essential in this setting. Submental flap reconstruction provided vascularized soft-tissue coverage in the immediate postoperative period while avoiding a more extensive reconstructive procedure.

## Introduction

Osteopetrosis comprises a rare and heterogeneous group of inherited bone disorders characterized by impaired osteoclastic bone resorption and increased bone density. Despite their radiographically dense appearance, affected bones are structurally abnormal and fragile, resulting in an increased risk of fractures and skeletal deformities. Defective bone remodeling may also contribute to dental abnormalities and disturbances in mineral homeostasis. In severe forms of the disease, excessive bone deposition can progressively obliterate the medullary cavities and narrow cranial nerve foramina, leading to bone marrow failure and neurological complications. Hematological manifestations may include anemia, thrombocytopenia, recurrent infections, bleeding tendency, and hepatosplenomegaly resulting from extramedullary hematopoiesis, while cranial nerve compression may cause visual and auditory impairment and other neurological deficits [1].

Osteopetrosis encompasses a broad clinical spectrum, ranging from asymptomatic or mildly symptomatic disease to severe forms presenting in infancy and associated with substantial morbidity and early mortality. Historically, the disease has been classified according to clinical severity and inheritance pattern into autosomal recessive, autosomal dominant, and intermediate forms. Autosomal recessive osteopetrosis generally represents the most severe phenotype, whereas autosomal dominant forms are often milder but may also result in clinically significant skeletal and hematological complications. The estimated incidence of osteopetrosis varies according to the clinical and genetic subtype, with historical estimates of approximately one in 200,000 live births for autosomal recessive osteopetrosis and one in 20,000 for autosomal dominant osteopetrosis [1-3].

Advances in molecular genetics have substantially improved the understanding and classification of osteopetrosis according to its underlying molecular mechanisms. Identification of the causative genetic variant may provide important information regarding disease phenotype and prognosis. However, considerable phenotypic heterogeneity may occur among individuals with osteopetrosis, including among patients carrying variants in the same gene [4]. Among the genes associated with osteopetrosis, CLCN7 is an important cause of both autosomal dominant and autosomal recessive forms of the disease. Pathogenic CLCN7 variants are associated with a broad clinical spectrum, ranging from relatively mild skeletal manifestations to severe disease, with considerable variability in clinical expression even among individuals from the same family [1,4].

Orofacial manifestations are an important component of osteopetrosis, and dental abnormalities may occur as part of the disease spectrum. Reported abnormalities include tooth agenesis, delayed or failed eruption, enamel hypoplasia, malformed crowns and roots, and premature tooth loss related to caries and other developmental disturbances [5,6]. Osteomyelitis is a well-recognized complication of osteopetrosis and most frequently affects the mandible. It may occur in association with odontogenic infection or dental procedures and can progress to exposed or necrotic bone. Treatment is challenging because of impaired bone remodeling, compromised local vascularity, and associated hematological abnormalities. Management generally relies on antimicrobial therapy and careful surgical debridement when necrotic bone is present [7,8].

The management of advanced mandibular osteomyelitis in patients with severe osteopetrosis represents a particular multidisciplinary challenge. Extensive bone sclerosis, impaired bone remodeling, compromised hematopoiesis, anemia, thrombocytopenia, and poor nutritional status may substantially increase the risks associated with surgical treatment and postoperative recovery. When necrotic bone and associated soft-tissue defects are present, treatment may require removal of nonviable tissue combined with reconstruction of the resulting defect. Perioperative management may additionally require optimization of hematological parameters, appropriate antimicrobial therapy, careful airway management, and intensive postoperative monitoring.

Reconstruction of anterior floor-of-mouth soft-tissue defects may involve local or regional pedicled flaps or free-tissue transfer, depending on the size and location of the defect and the patient's general condition. In medically compromised pediatric patients, the choice of reconstructive technique must also take into account operative burden, donor-site morbidity, vascular reliability, and the patient's ability to tolerate prolonged or extensive surgery. In this context, the submental flap represents a potential option for vascularized soft-tissue coverage while avoiding the greater operative burden associated with free-tissue transfer.

Here, we present the case of a 14-year-old girl with severe osteopetrosis, reported in her previous medical records as CLCN7-related, complicated by hematological abnormalities, hepatosplenomegaly, neurological complications, and chronic mandibular osteomyelitis with necrotic bone. The patient underwent removal of the necrotic mandibular bone with simultaneous reconstruction of the anterior floor-of-mouth soft-tissue defect using a submental flap, followed by postoperative intensive care management. This case illustrates the surgical, hematological, perioperative, and postoperative challenges associated with the treatment of advanced mandibular disease in severe osteopetrosis.

## Case presentation

A 14-year-old girl with severe osteopetrosis, reported in her previous medical records as CLCN7-related, was referred to our tertiary center because of severe mandibular complications. According to the medical history and documentation from the center responsible for her previous care, molecular genetic testing had identified two variants in the CLCN7 gene. The original genetic report was not available to the authors; therefore, the exact variant nomenclature, zygosity, phase, segregation data, testing methodology, and classification could not be independently verified.

The patient was born at approximately 36 weeks of gestation by cesarean section because of fetal compromise associated with maternal hypertension, fetal growth restriction, and oligohydramnios. Her birth weight was 1740 g, and her Apgar score was 10 points. At seven months of age, communicating hydrocephalus, most likely posthemorrhagic, was diagnosed in the setting of increased intracranial pressure. At eight months of age, a ventriculoperitoneal shunt was implanted, and the patient remained under regular neurosurgical follow-up. Her medical history also included long-bone fractures, optic nerve atrophy, hypothyroidism, delayed psychomotor development, previous surgical treatment of a floor-of-mouth phlegmon, and clinically evident cachexia with reduced muscle mass. Her regular medications included levothyroxine 37.5 μg daily, vitamins B1, B6, B12, C, D, and folic acid.

The hematological manifestations of osteopetrosis were severe. According to the available records from a previous hospitalization in a pediatric hematology department, bone marrow aspiration was unsuccessful, while trephine biopsy was technically difficult but ultimately obtained. Histopathological examination demonstrated marked obliteration of the intertrabecular spaces by thickened bone trabeculae, with an absence of normal hematopoietic marrow. Significant splenomegaly was also documented in the context of suspected extramedullary hematopoiesis. Reticulocytosis of 4% was documented, while an eosin-5-maleimide (EMA) test was negative. Elevated D-dimer concentrations were reported in the available medical records; however, the underlying cause could not be independently established from the available documentation. According to the previous medical assessment, hematopoietic stem cell transplantation had been considered but was not pursued because of the advanced stage of disease and the patient's clinical condition. She subsequently required hospitalization in a pediatric hematology and oncology department because of anemia and received transfusions of blood products.

According to the history obtained from the patient's mother, symptoms of mandibular inflammation and recurrent abscess formation had been present for approximately three years before admission to our institution. During this period, the patient required repeated hospitalizations at different medical centers. During the most recent hospitalization, the mandibular abscess was incised and drained, and necrotic tissues were debrided. The patient was subsequently referred to our Department of Maxillofacial Surgery. Following imaging studies, specialist consultations, and hematological preparation, removal of necrotic mandibular lesions with simultaneous submental flap reconstruction was performed.

On admission to the Department of Maxillofacial Surgery, the patient was in a relatively stable general condition but presented with marked features of advanced osteopetrosis. Physical examination revealed significant short stature, cachexia, reduced muscle mass, and a grayish skin complexion. There was proptosis and visual impairment. A ventriculoperitoneal shunt was visible over the left parietal-occipital region. Marked splenomegaly was present, with the spleen extending toward the left iliac fossa, accompanied by hepatomegaly of approximately 3-4 cm below the right costal margin. Contractures of the lower extremity joints were also noted.

On admission, laboratory evaluation demonstrated anemia and thrombocytopenia, with a hemoglobin concentration of 8.4 g/dL and a platelet count of 61 × 10⁹/L, as shown in Table 1. A pediatric hematology consultation recommended additional laboratory evaluation, abdominal ultrasonography, intravenous vitamin K administration, and optimization of hematological parameters before surgery. Following hematological optimization and transfusion of blood products, the hemoglobin concentration increased to 11.7 g/dL, and the platelet count to 83 × 10⁹/L. The patient received two units of packed red blood cells and two units of platelet concentrate before surgery. Subsequent preoperative laboratory testing is presented in Table 2. Additional preoperative abnormalities included increased red cell distribution width (RDW), mild leukocytosis with an elevated proportion of immature granulocytes, an elevated CRP concentration of 29 mg/L (reference range <5 mg/L), and a markedly elevated D-dimer level of 5368.2 ng/mL. The activated partial thromboplastin time was within the laboratory reference range, while fibrinogen was reported as within the normal range in the available medical records.

**Table 1:** Abnormal laboratory findings on admission.

Laboratory parameter	Patient value	Reference range	Unit
Hemoglobin (Hgb)	8.4	11.8-14.8	g/dL
Platelet count (PLT)	61	150-388	×10⁹/L
International normalized ratio (INR)	1.4	0.9-1.3	-

**Table 2:** Preoperative laboratory findings. Abbreviations: APTT, activated partial thromboplastin time; CRP, C-reactive protein; IG, immature granulocytes; INR, international normalized ratio; MCH, mean corpuscular hemoglobin; MCHC, mean corpuscular hemoglobin concentration; MCV, mean corpuscular volume; PT, prothrombin time; RBC, red blood cell count; RDW-CV, red cell distribution width-coefficient of variation; RDW-SD, red cell distribution width-standard deviation; TT, thrombin time; WBC, white blood cell count

Parameter	Result	Reference range	Unit
Hemoglobin	11.7	11.8-14.8	g/dL
Hematocrit	36.8	36-46	%
RBC	4.5	4.1-5.1	×10⁶/mm³
MCV	81	81.1-95.6	fL
MCH	26	25.7-31.7	pg
MCHC	31.8	31.9-33.8	g/dL
RDW-CV	18.5	12-14	%
RDW-SD	54.5	37.4-44.7	fL
Platelets	65	150-388	×10³/mm³
WBC	10.1	4-9.2	×10³/mm³
Neutrophils	6.5	1.6-6.5	×10³/mm³
Neutrophils	64.4	37.5-72.8	%
Lymphocytes	2.9	1.41-3.65	×10³/mm³
Lymphocytes	28.4	20.6-55.9	%
Monocytes	0.6	0.22-0.62	×10³/mm³
Monocytes	5.5	3.9-9.9	%
Eosinophils	0.2	0.04-0.59	×10³/mm³
Eosinophils	1.5	0.7-9.3	%
Basophils	0	0.02-0.03	×10³/mm³
Basophils	0.2	0.1-0.6	%
Immature granulocytes (IG)	1.85	0.1-1.3	×10³/mm³
Immature granulocytes (IG%)	18.3	<0.01	%
CRP	29	<5	mg/L
PT	13.7	12.1-14.6	s
APTT	31.7	25.1-40	s
INR	1.25	0.9-1.3	-
Thrombin time (TT)	18.3	14-21	s
D-dimer	5368.2	<500	ng/mL
Sodium	139.9	132-141	mmol/L
Potassium	3.6	3.5-5.1	mmol/L
Chloride	104.6	98-107	mmol/L
Urine specific gravity	1.020	1.012-1.026	-
Urine pH	6.5	4-6.8	-

Computed tomography angiography of the craniofacial region demonstrated generalized marked thickening and sclerosis of the examined bones, consistent with advanced osteopetrosis. Significant bone defects were present in the anterior mandibular body, with adjacent air bubbles interpreted as post-inflammatory and post-surgical changes, as shown in Figure 1.

**Figure 1:**
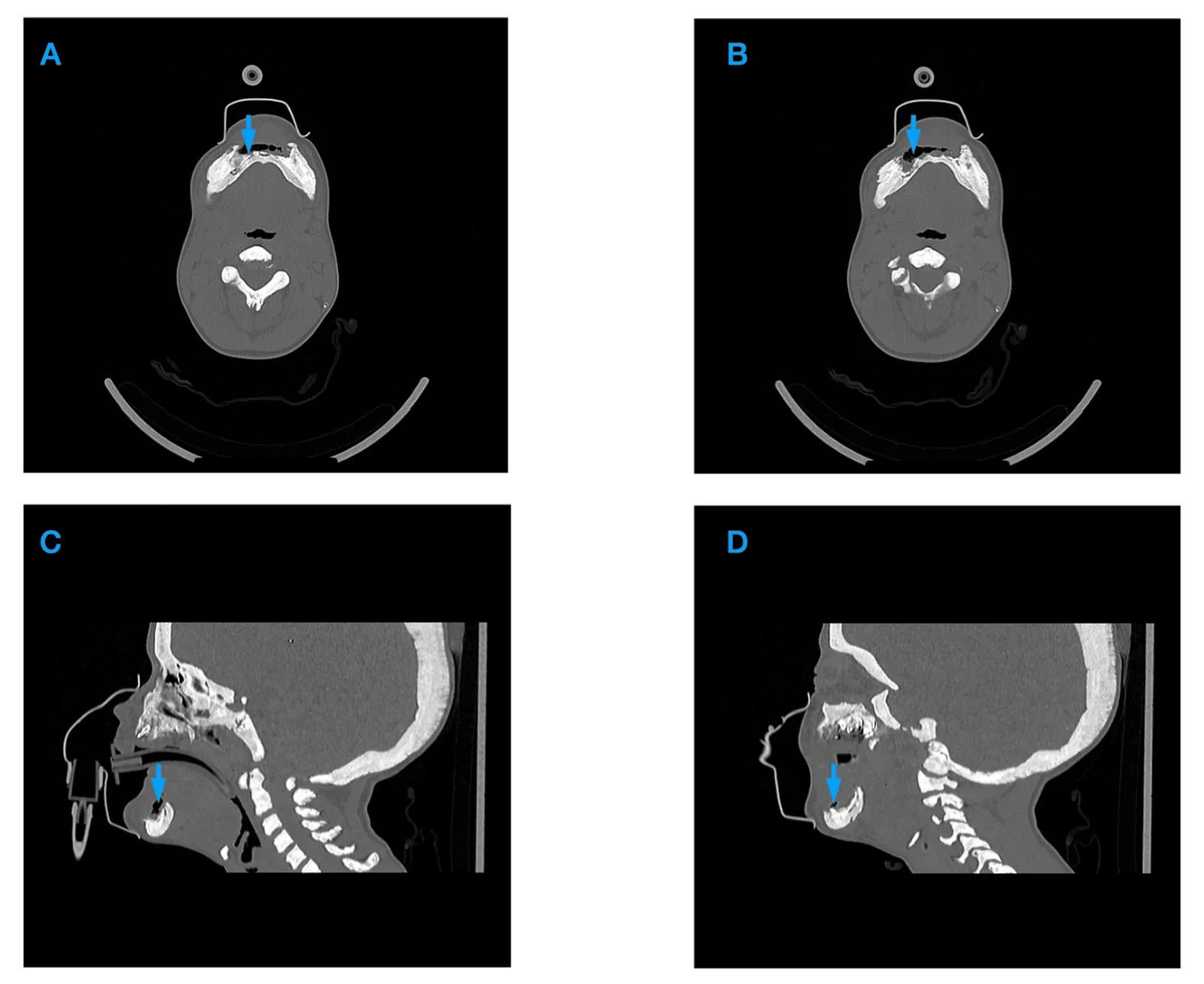
Preoperative computed tomography (CT) images demonstrating pathological changes of the anterior mandible. (A, B) Axial CT images showing marked sclerosis and defects involving the anterior mandibular body, with adjacent air collections (arrows), consistent with the described post-inflammatory and post-surgical changes. (C, D) Sagittal CT images demonstrating extensive sclerotic changes and areas of osseous destruction involving the anterior mandible (arrows).

Both submental arteries were patent throughout their course, and both facial arteries demonstrated contrast enhancement along their entire visualized course, as shown in Figure 2.

**Figure 2:**
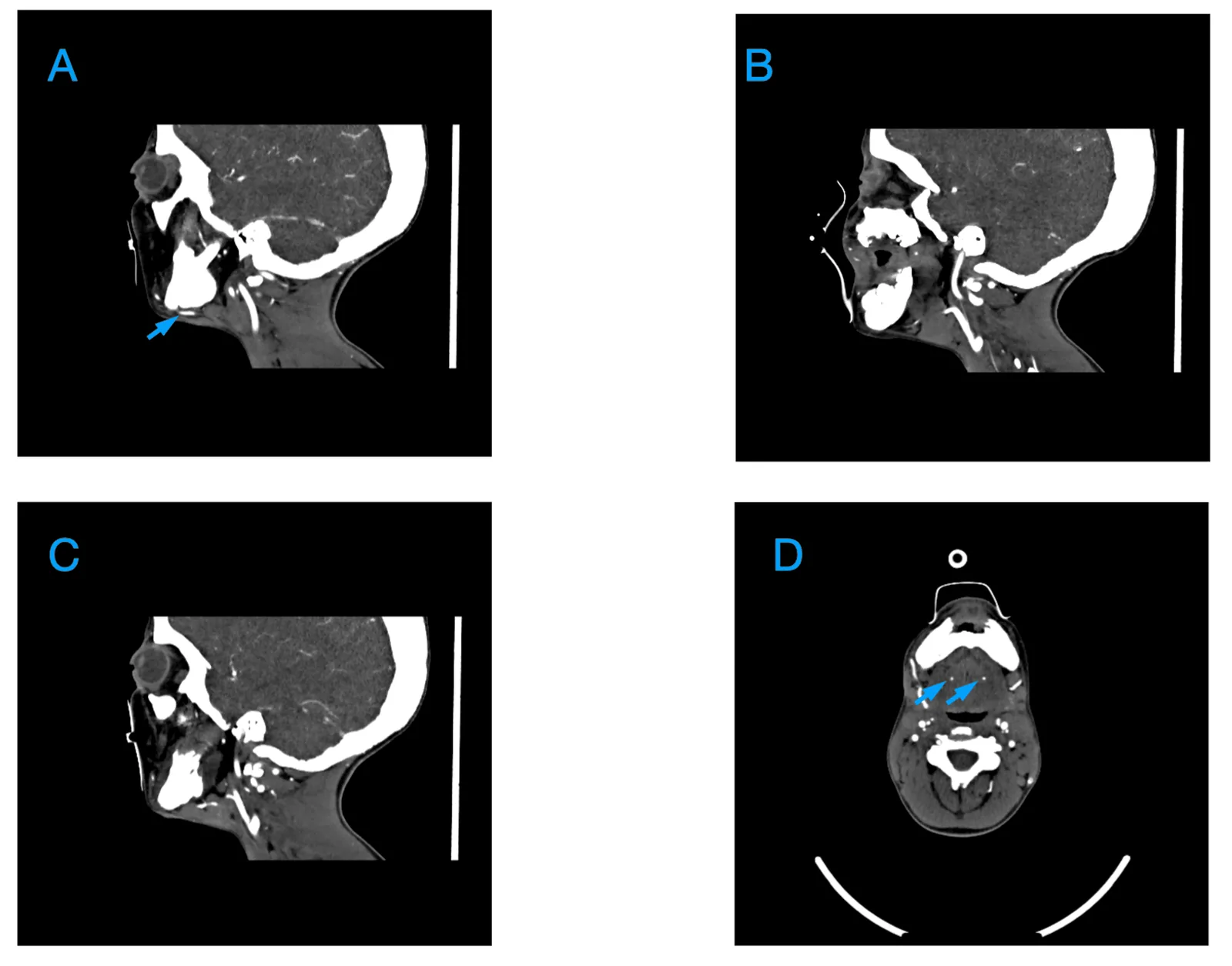
Computed tomography angiography demonstrating patency of the submental arteries. (A-C) Sagittal images. (D) Axial image. Arrows indicate the patent submental arteries.

Abdominal ultrasonography demonstrated marked splenomegaly, with the spleen measuring 189 × 95 mm and containing additional splenic tissue measuring approximately 18 mm, as shown in Figure 3. The liver was mildly enlarged, extending approximately 28 mm below the right costal margin, with preserved parenchymal echogenicity. A small amount of free fluid was observed in the perisplenic region and the pelvic recess.

**Figure 3:**
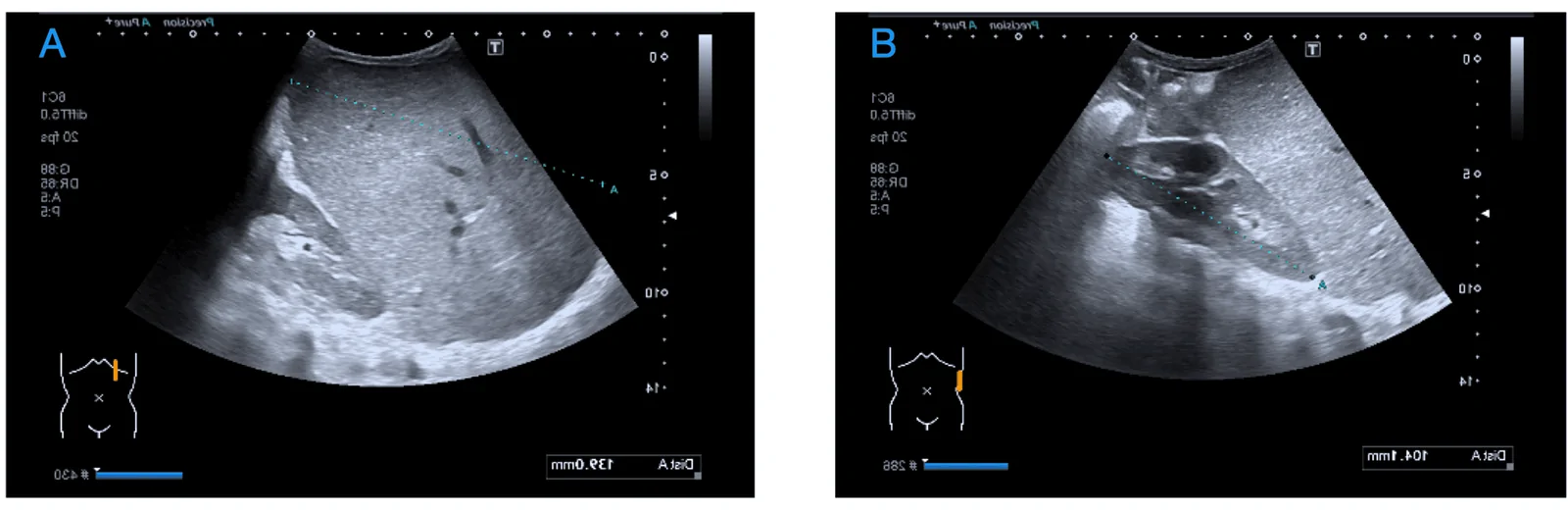
(A, B) Abdominal ultrasonography demonstrating marked splenomegaly, with the spleen measuring 189 × 95 mm.

Following induction of anesthesia, oral endotracheal intubation was performed using videolaryngoscopy, with placement of a size 6 endotracheal tube. The patient subsequently underwent surgical treatment of the mandibular lesion, including intraoral osteotomy and removal of necrotic mandibular bone with debridement of the affected tissues. Simultaneous reconstruction of the anterior floor of the mouth was performed using a submental flap. The flap was harvested through a submental incision and included the platysma and anterior belly of the digastric muscle. The facial vessels were identified and preserved, and the submandibular gland was mobilized. Following intraoral debridement and preparation of the mucosal margins, a tunnel was created through the floor of the mouth, and the flap was transferred intraorally. The skin paddle was positioned within the tissue defect and securely sutured.

The procedure did not involve segmental mandibular resection; the necrotic mandibular bone was removed by intraoral osteotomy and debridement, without intentional interruption of mandibular continuity. No bony reconstruction or fixation was required

Histopathological examination of the submandibular lymph node demonstrated reactive changes, with preserved follicular architecture, reactive lymphoid follicles, and sinus histiocytosis. Histopathological examination of the submandibular gland demonstrated periductal and interlobular fibrosis with scattered individual lymphocytes.

The clinical course of the patient, from the systemic manifestations of CLCN7-related osteopetrosis to mandibular involvement, surgical treatment, and postoperative management, is summarized in Figure 4.

**Figure 4:**
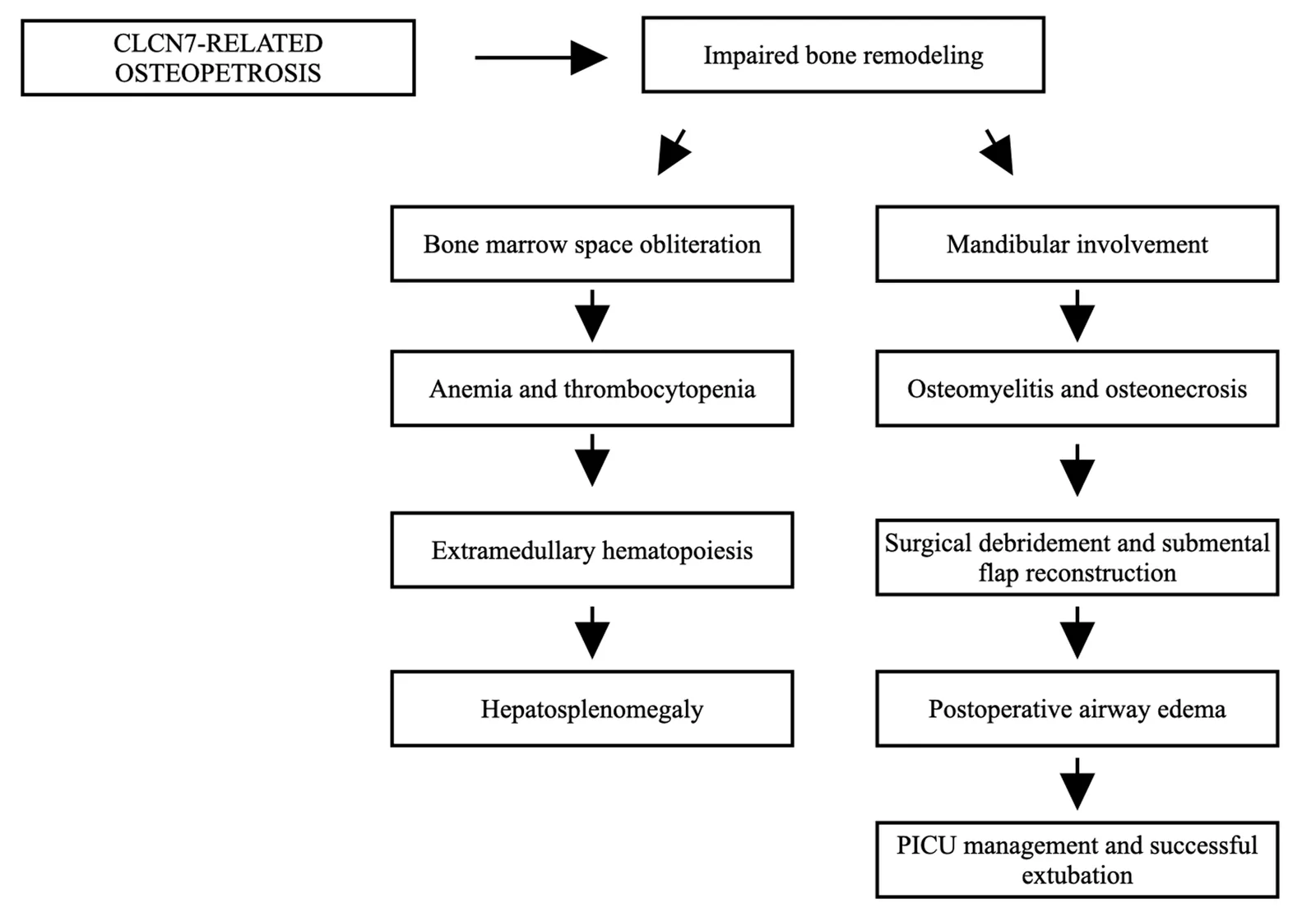
Clinical course and management of CLCN7-related osteopetrosis complicated by mandibular osteomyelitis.

Postoperatively, the patient developed significant soft-tissue edema with concern for upper airway obstruction and was therefore transferred to the pediatric intensive care unit (PICU) while intubated and mechanically ventilated. Oral endotracheal intubation had been performed using videolaryngoscopy, with placement of a size 6 endotracheal tube. On admission to the PICU, she remained under analgosedation. Physical examination demonstrated marked cachexia and reduced muscle mass, grayish skin coloration, proptosis, and pronounced hepatosplenomegaly. A postoperative neck dressing and Redon drain were present, with only a small amount of blood in the drain.

The preoperative intraoral appearance and the early postoperative result following removal of the necrotic mandibular bone and reconstruction with a submental flap are shown in Figure 5.

**Figure 5:**
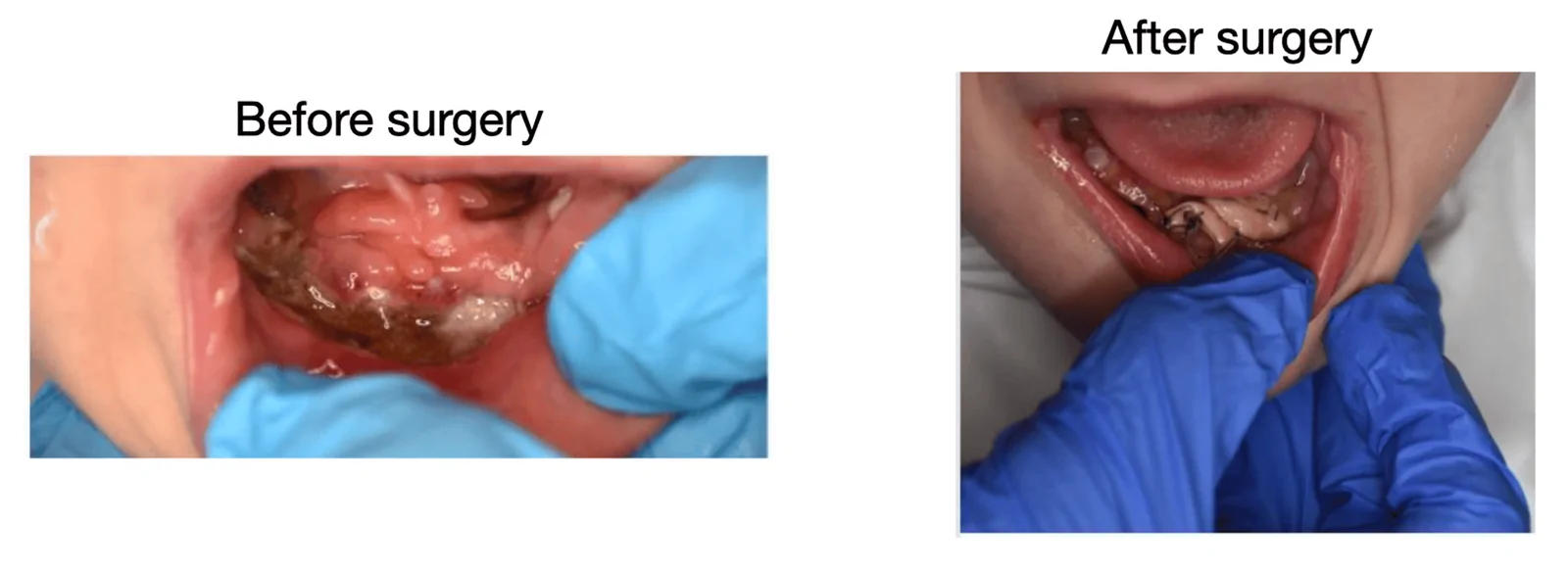
Clinical appearance of the mandibular and anterior floor-of-mouth region before and after surgery. (A) Preoperative intraoral view showing the affected anterior mandibular region with necrotic tissue. (B) Postoperative intraoral view following removal of the necrotic mandibular bone and reconstruction of the anterior floor-of-mouth soft-tissue defect using a submental flap.

Analgesia and sedation were maintained with midazolam and sufentanil, with ketamine also used perioperatively. Mechanical ventilation was continued using synchronized intermittent mandatory ventilation with pressure control (SIMV-PC mode) with an initial FiO₂ of 0.30, with ventilatory parameters adjusted according to serial blood-gas analyses. Cardiovascular status remained stable, with a tendency toward mild hypertension managed pharmacologically. Fluid therapy was adjusted according to the patient's clinical status, with diuresis supported by fractionated doses of furosemide. Enteral nutritional support with Nutrini was provided during the perioperative period. In accordance with the pediatric hematology recommendations, deferoxamine was administered to reduce the risk of excessive iron accumulation associated with repeated blood transfusions. Antibiotic therapy was initially administered empirically and was subsequently adjusted according to antimicrobial susceptibility results. During the perioperative period, amoxicillin/clavulanic acid and metronidazole were continued.

On the third postoperative day, sedation was discontinued, and the patient was successfully extubated. She subsequently maintained spontaneous breathing with passive oxygen therapy. Her respiratory and cardiovascular status remained stable, and the postoperative edema gradually decreased. The surgical wounds demonstrated satisfactory early healing without documented acute postoperative complications.

After stabilization, the patient was transferred from the PICU to the Department of Head and Neck Surgery for Children and Adolescents for continued postoperative care. The available documentation covers the early postoperative period; long-term clinical and radiological follow-up was not available.

## Discussion

The present case illustrates the complexity of managing severe osteopetrosis, reported in the patient's previous medical records as CLCN7-related, complicated by chronic mandibular osteomyelitis and significant hematological dysfunction. The patient presented with anemia, marked hepatosplenomegaly, neurological complications, and extensive skeletal involvement, reflecting the severe multisystem clinical manifestations of her disease [1,7].

Hematological complications are among the major manifestations of severe osteopetrosis. Progressive narrowing and obliteration of the medullary cavities impair normal hematopoiesis and may result in anemia, thrombocytopenia, and compensatory extramedullary hematopoiesis, frequently manifesting as hepatosplenomegaly [1,7,9]. In our patient, trephine biopsy demonstrated marked replacement of the marrow spaces by abnormal bone trabeculae, providing a morphological explanation for the severe hematological abnormalities. The cytopenias had direct implications for surgical management and required preoperative transfusion support and close perioperative hematological monitoring. Hematopoietic stem cell transplantation may represent a potentially curative treatment for selected patients with severe forms of osteopetrosis, although its applicability depends on the underlying molecular subtype, clinical condition, and presence of established complications [7,10]. In the present patient, transplantation had previously been considered but was ultimately not pursued.

Mandibular osteomyelitis is a particularly challenging complication of osteopetrosis. It most commonly affects the mandible and may develop following odontogenic infection or dental procedures, with impaired bone remodeling and vascular compromise contributing to persistent infection and poor healing [11-15]. In our patient, mandibular infection had a chronic and recurrent course, with repeated abscess formation and previous surgical drainage. The coexistence of extensive mandibular osteosclerosis and severe systemic disease further increased the risks associated with surgical treatment.

The optimal extent of surgery in osteopetrosis-associated mandibular osteomyelitis remains uncertain. Reported treatment strategies include antimicrobial therapy, sequestrectomy, conservative debridement, and, in refractory cases, partial or segmental mandibular resection [11-14]. More extensive resection may facilitate removal of affected bone but can result in substantial functional and reconstructive morbidity, particularly in patients with severe systemic disease [13,14]. Conversely, conservative surgery may be associated with persistent or recurrent infection. Therefore, the surgical approach should be individualized according to the extent of necrotic bone, severity of infection, systemic condition, and potential for adequate soft-tissue healing [12,15].

In the present case, avoiding segmental mandibular resection was an important treatment consideration. Given the patient's anemia, thrombocytopenia, clinically evident cachexia, and limited physiological reserve, extensive mandibular resection was considered potentially associated with substantial morbidity. Limited removal of the documented necrotic mandibular bone was therefore performed, followed by immediate reconstruction of the associated anterior floor-of-mouth soft-tissue defect with a submental flap. The flap was selected because it provided vascularized soft-tissue coverage while avoiding the additional operative burden associated with free-tissue transfer in a patient with severe systemic disease. Preoperative CT imaging demonstrated patency of the relevant facial and submental vascular structures, supporting the feasibility of the planned reconstruction. The procedure did not involve segmental mandibular resection, and mandibular continuity was not intentionally interrupted. However, because postoperative radiological imaging was not available, preservation of mandibular continuity could not be independently confirmed radiologically. The submental flap was used for soft-tissue reconstruction and not for bony mandibular reconstruction. This approach represents the individualized management strategy used in our patient rather than evidence that it is superior to other reconstructive techniques.

The postoperative course further demonstrates the importance of multidisciplinary perioperative management in patients with severe osteopetrosis. Postoperative airway edema should be considered multifactorial. In addition to the patient's systemic disease and limited physiological reserve, local factors related to flap bulk, submental dissection, tissue manipulation, and possible venous congestion may have contributed to postoperative swelling. Because the available documentation does not allow these factors to be individually assessed, no specific causal attribution can be made. The patient required mechanical ventilation and pediatric intensive care, with management including analgesia and sedation, antimicrobial therapy, fluid and diuretic management, hematological support, nutritional support, and close respiratory monitoring. Successful extubation was achieved on the third postoperative day, followed by clinical stabilization and transfer to the maxillofacial surgical ward. The potential for postoperative airway compromise should therefore be considered during planning of similar reconstructions. However, this single case does not provide sufficient evidence to recommend routine planned tracheostomy or a specific alternative flap inset strategy.

This report describes a single patient and therefore cannot establish the superiority of the surgical or reconstructive approach used. In addition, the available documentation covers only the immediate and early postoperative period, limiting conclusions regarding long-term flap status, recurrence of mandibular osteomyelitis, and radiological outcomes. Nevertheless, the case illustrates the challenges of surgical management in severe osteopetrosis and emphasizes the importance of individualized treatment planning and multidisciplinary perioperative care.

## Conclusions

Severe osteopetrosis complicated by mandibular osteomyelitis and significant hematological dysfunction represents a major therapeutic challenge requiring individualized multidisciplinary management. In our patient, limited removal of the documented necrotic mandibular bone was combined with immediate vascularized soft-tissue reconstruction of the anterior floor-of-mouth defect using a submental flap. Careful preoperative hematological optimization, vascular assessment, antimicrobial therapy, nutritional support, and postoperative intensive care were integral to the perioperative management.

This case emphasizes that management of severe osteopetrosis should be tailored to the patient's systemic condition, extent of osseous disease, and reconstructive possibilities. The documented early postoperative course included satisfactory wound healing, reduction of postoperative edema, successful extubation on the third postoperative day, and subsequent clinical stabilization. However, the absence of long-term clinical and radiological follow-up limits conclusions regarding long-term infection control, flap outcomes, and mandibular status.
